# Relationship between the Rod complex and peptidoglycan structure in *Escherichia coli*

**DOI:** 10.1002/mbo3.1385

**Published:** 2023-10-09

**Authors:** Risa Ago, Yuhei O. Tahara, Honoka Yamaguchi, Motoya Saito, Wakana Ito, Kaito Yamasaki, Taishi Kasai, Sho Okamoto, Taiki Chikada, Taku Oshima, Issey Osaka, Makoto Miyata, Hironori Niki, Daisuke Shiomi

**Affiliations:** ^1^ Department of Life Science, College of Science Rikkyo University Tokyo Japan; ^2^ Graduate School of Science Osaka Metropolitan University Osaka Japan; ^3^ The OMU Advanced Research Center for Natural Science and Technology Osaka Metropolitan University Osaka Japan; ^4^ Department of Biotechnology, Faculty of Engineering Toyama Prefectural University Imizu Toyama Japan; ^5^ Department of Pharmaceutical Engineering, Faculty of Engineering Toyama Prefectural University Imizu Toyama Japan; ^6^ Microbial Physiology Laboratory, Department of Gene Function and Phenomics National Institute of Genetics Mishima Shizuoka Japan; ^7^ Department of Genetics The Graduate University for Advanced Studies, SOKENDAI Mishima Shizuoka Japan

**Keywords:** cell shape, peptidoglycan, Rod complex, suppressor mutation

## Abstract

Peptidoglycan for elongation in *Escherichia coli* is synthesized by the Rod complex, which includes RodZ. Although various mutant strains of the Rod complex have been isolated, the relationship between the activity of the Rod complex and the overall physical and chemical structures of the peptidoglycan have not been reported. We constructed a RodZ mutant, termed RMR, and analyzed the growth rate, morphology, and other characteristics of cells producing the Rod complexes containing RMR. The growth and morphology of RMR cells were abnormal, and we isolated suppressor mutants from RMR cells. Most of the suppressor mutations were found in components of the Rod complex, suggesting that these suppressor mutations increase the integrity and/or the activity of the Rod complex. We purified peptidoglycan from wild‐type, RMR, and suppressor mutant cells and observed their structures in detail. We found that the peptidoglycan purified from RMR cells had many large holes and different compositions of muropeptides from those of WT cells. The Rod complex may be a determinant not only for the whole shape of peptidoglycan but also for its highly dense structure to support the mechanical strength of the cell wall.

## Graphical abstract

The structures of the peptidoglycan of the wild‐type strain, the mutant strain of the Rod complex, and its suppressor mutant strains were observed. The peptidoglycan purified from the Rod complex mutant had many large holes, whereas the number and size of holes were reduced in the suppressor mutants. We visualized the relationship between the Rod complex activity and the peptidoglycan structure.

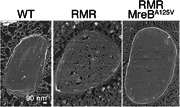

## 1 INTRODUCTION

**Figure 1:**
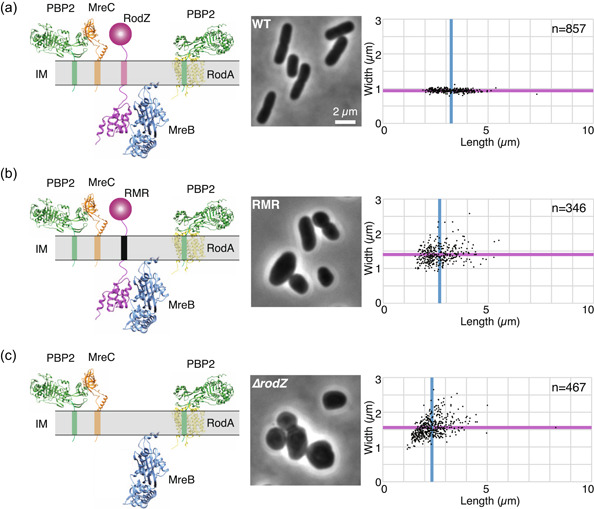
Morphology of cells producing mutant Rod complex. (a–c) Schematic illustrations of Rod complex containing WT RodZ (a, left) or RMR (b, left) and Rod complex without RodZ (∆*rodZ*) (c, left). For the structure of each protein, we used the structures registered in the database (https://alphafold.ebi.ac.uk) of proteins predicted by Alpha Fold2 (Jumper et al., 2021). IM, inner membrane. Morphology of cells producing WT RodZ (a, middle and right) or RMR (b, middle and right) and cells lacking *rodZ* (c, middle and right) and distribution of length and width of each strain (right). Phase contrast images are shown (middle). Blue and magenta lines indicate the average length and width of WT cells, respectively.

Bacterial cells show a wide variety of cell shapes, such as round, rod, and spiral (Young, 2003; Young, 2010). Each bacterial species has to maintain its shape during various cellular events, including cell division and segregation of genomic DNA. Most bacterial cells are surrounded by peptidoglycan, a macromolecule consisting of glycan strands crosslinked by short peptides. Peptidoglycan determines cell shape because the shape of the purified peptidoglycan is reminiscent of that of the bacterial cells (Egan et al., 2020; Pedro et al., 1997; Rohs & Bernhardt, 2021). *Escherichia coli* exhibits a rod shape consisting of a central cylinder and polar caps. The synthesis of peptidoglycan is regulated by the Rod complex (Figure 1a), including actin homolog MreB, peptidoglycan synthases penicillin‐binding protein (PBP) 2 and RodA, a transmembrane protein RodZ, MreC, and MreD (Ago & Shiomi, 2019; Blaauwen et al., 2008; Egan et al., 2020; Rohs & Bernhardt, 2021). PBP2 is a transpeptidase required for cell elongation (Sauvage et al., 2008; Spratt, 1975) and RodA is a glycosyltransferase (Emami et al., 2017; Meeske et al., 2016; Sjodt et al., 2020). The *mreC* and *mreD* genes constitute an operon with the *mreB* gene, and these gene products are functionally related. MreC interacts with MreB and MreD, whereas MreD does not interact with MreB (Kruse et al., 2005). MreC also interacts with PBP2 (Contreras‐Martel et al., 2017) and this interaction is thought to cause a structural change in PBP2 and stimulate peptidoglycan polymerization and crosslinking (Rohs et al., 2018). It has been shown that the balance between MreC and MreD determines the activity of PBP2 (Liu et al., 2020). RodZ physically and genetically interacts with itself, MreB, MreC, MreD, PBP2, and RodA (Bendezú et al., 2009; Ikebe et al., 2018; Morgenstein et al., 2015; Shiomi et al., 2008, 2013). Thus, RodZ interacts with all known major components of the Rod complex and therefore plays a key role in this complex. RodZ forms a “superstructure” of high molecular weight which dissociates into a hexamer, suggesting that the Rod complex consists of several small units including the RodZ hexamer (Mitobe et al., 2020). The Rod complex is highly dynamic; that is, the Rod complex rotates perpendicularly to the long axis of the cell (Domínguez‐Escobar et al., 2011; Garner et al., 2011; Teeffelen et al., 2011), allowing the insertion of peptidoglycan in the cell surface layer in an evenly distributed manner. Therefore, if the presence of the Rod complex components and the interactions between the components are not maintained correctly, the peptidoglycan will not be formed correctly, resulting in abnormal morphology. Such morphological abnormalities can cause growth inhibition and cell death.

The transmembrane protein RodZ is not essential for viability but is critical for cell shape maintenance and fast growth in *E. coli* (Bendezú et al., 2009; Shiomi et al., 2008). Cells lacking *rodZ* are round or oval in shape and grow slower than WT cells. We previously isolated mutants that suppressed the *rodZ* phenotypes and found that most of the mutations occurred in *mreB*, *mrdA* (encoding PBP2), and *mrdB* (encoding RodA) (Shiomi et al., 2013). Most of the mutations in *mreB* are located at the interface between two MreB filaments, so these mutations would strengthen MreB assembly without RodZ, suggesting that RodZ helps in the assembly of MreB filaments in vivo. One of the suppressor mutations (RodA^A234T^) found in *mrdB* was shown to have an increased activity of peptidoglycan synthesis (Rohs et al., 2018). Interestingly, cells producing these suppressor mutants, or PBP2^L61R^ which suppressed *mreC* defective mutants, were resistant to A22, which inhibits MreB assembly (Rohs et al., 2018; Shiomi et al., 2013), suggesting that MreB filament is more stable in these suppressor strains than in WT cells. It is unclear how the effects or signals of mutations in PBP2 or RodA are transmitted to MreB, or what proteins are involved in the process. One of the candidate proteins is RodZ because it interacts with MreB, PBP2, and RodA. In particular, because MreB is a cytoplasmic protein and the active sites of PBP2 and RodA are in the periplasm, the transmembrane domain of RodZ appears to be important for the transmission or connection between MreB and PBP2/RodA through RodZ (Morgenstein et al., 2015).

The chemical structure of peptidoglycan and its synthetic pathways have been studied for many years. The structure of peptidoglycan was visualized using electron microscopy (EM) and atomic force microscopy (AFM). These observations revealed the meshwork structure of peptidoglycan and the arrangement of glycan strands perpendicular to the long axis (de Pedro et al., 1997; Gan et al., 2008; Pasquina‐Lemonche et al., 2020; Tulum et al., 2019; Turner et al., 2018). Recently, it was shown by AFM that treatments of *E. coli* with β‐lactam and *Staphylococcus aureus* with antibiotics such as methicillin and vancomycin result in holes in the peptidoglycan (Elsbroek et al., 2023; Salamaga et al., 2021). If the balance between peptidoglycan synthesis and hydrolysis is not properly maintained, the peptidoglycan structure cannot be maintained and bacterial cells would be lysed. The relationship between the overall structure of the peptidoglycan and the activity of the Rod complex is unclear.

To investigate the relationship between the activity of the Rod complex and the structure of peptidoglycan, we constructed and characterized a chimeric protein of RodZ and MalF, named RMR, in which the transmembrane domain of RodZ was replaced with the corresponding domain of MalF. Cells producing RMR grew slower than WT cells and showed an abnormal shape. The subcellular localization of RMR was different from that of WT RodZ. We isolated suppressor mutations of the slow growth phenotype of RMR, and the suppressors restored rod shape and the localization of the Rod complex containing RMR was rescued to a WT Rod complex localization pattern. Most of the mutations were mapped to components of the Rod complex. We then directly observed peptidoglycan by quick‐freeze, deep‐etch electron microscopy(QFDE‐EM). This method is suitable for observing the bacterial cell surface layer with high resolution (Ojima et al., 2021; Tulum et al., 2019). In particular, the structure of the surface layer (peptidoglycan layer) of *Bacillus subtilis* and its L‐form cells was recently observed with this method (Tulum et al., 2019). Using these methods, we found that peptidoglycan purified from cells producing RMR had more and larger holes than the suppressors. We also analyzed the chemical structures of muropeptide and found that the suppressor mutation certainly restored the chemical structure of muropeptide from RMR‐type to WT.

## 2 MATERIALS AND METHODS

### 2.1 Bacterial strains and growth medium

All strains were derivatives of *E. coli* K‐12 and are listed in Table 1. BW25113 is a wild‐type strain (Baba et al., 2006), RU2 (∆*rodZ::kan*) lacks the *rodZ* gene and is a derivative of BW25113 (Ikebe et al., 2018; Shiomi et al., 2008). Cells were grown in L broth (1% Bacto tryptone, 0.5% yeast extract, 0.5% NaCl) at 37°C. Kanamycin (Kan; 50 µg mL^−1^), ampicillin (Amp; 100 µg mL^−1^), and chloramphenicol (Cm; 20 µg mL^−1^) were added to the culture medium when necessary. The absorbance (OD_660_) was measured every 5 min using a compact rocking incubator (TVS062CA; ADVANTEC).

**Table 1:** Strains used in this study.

Strain	Relevant genotype	References
BW25113	WT	Baba et al. (2006)
RU2	BW25113 ∆*rodZ*::kan	Shiomi et al. (2008)
RU383	BW25113 *sfgfp‐rodZ*	Ikebe et al. (2018)
RU386	BW25113 *sfgfp‐rodZ mreB‐mCherry* ^*SW*^	Yoshii et al. (2019)
RU1353	BW25113 *sfgfp‐rmr*	This study
RU1354	BW25113 *sfgfp‐rmr mreB‐mCherry* ^*SW*^	This study
RU1477	the original suppressor 2‐4 (*mreB* ^*A125V*^)	This study
RU1482	the original suppressor 4‐1 (*mreB* ^*R124S*^)	This study
RU1483	the original suppressor 4‐2 (*mrdB* ^*A234T*^)	This study
RU1484	the original suppressor 4‐3 (*mreB* ^*E137G*^)	This study
RU1485	the original suppressor 4‐5 (*mreC* ^*S153I*^)	This study
RU1486	the original suppressor 4‐6 (*mreD* ^*F123L*^)	This study
RU1487	the original suppressor 4‐7 (*mrdA* ^*R234L*^)	This study
RU1488	the original suppressor 4‐8 (*mrdA* ^*T52I*^)	This study
RU1490	the original suppressor 4‐13 (*mrdA* ^*I59S*^)	This study
RU1491	the original suppressor 4‐14 (*mrdB* ^*K243N*^)	This study
RU1492	the original suppressor 4‐15 (*mreB* ^*E122D*^)	This study
RU1493	the original suppressor 4‐16 (*mreB* ^*R124L*^)	This study
RU1495	the original suppressor 4‐19 (*mrdA* ^*A201V*^)	This study
RU1496	the original suppressor 4‐20 (*mrdA* ^*V227L*^)	This study
RU1701	RU1492 (*mreB* ^*E122D*^) ∆*yhdE*::cat	This study
RU1647	RU1493 (*mreB* ^*R124L*^) ∆*yhdE*::cat	This study
DS1157	BW25113 *mreB* ^*R124S*^ ∆*yhdE*::cat	Shiomi et al. (2013)
DS612	BW25113 *mreB* ^*A125V*^ ∆*yhdE*::cat	Shiomi et al. (2013)
RU1640	RU1484 (*mreB* ^*E137G*^) ∆*yhdE*::cat	This study
RU1641	RU1485 (*mreC* ^*S153I*^) ∆*yhdE*::cat	This study
RU1642	RU1486 (*mreD* ^*F123L*^) ∆*yhdE*::cat	This study
RU1644	RU1488 (*mrdA* ^*T52I*^) ∆*rlpA*::cat	This study
RU1645	RU1490 (*mrdA* ^*I59S*^) ∆*rlpA*::cat	This study
RU1702	RU1495 (*mrdA* ^*A201V*^) ∆*rlpA*::cat	This study
RU1648	RU1496 (*mrdA* ^*V227L*^) ∆*rlpA*::cat	This study
RU1643	RU1487 (*mrdA* ^*R234L*^) ∆*rlpA*::cat	This study
DS686	BW25113 *mrdB* ^*A234T*^ ∆*rlpA*::cat	Shiomi et al. (2013)
RU1646	RU1491 (*mrdB* ^*K243N*^) ∆*rlpA*::cat	This study
DS452	BW25113 ∆*yhdE::cat*	Shiomi et al. (2013)
DS454	BW25113 ∆*rodZ::kan* ∆*yhdE::cat*	Shiomi et al. (2013)
RU1716	BW25113 *sfgfp‐rodZ* ∆*yhdE::cat mreB* ^*E122D*^	This study
RU1721	BW25113 *sfgfp‐rmr* ∆*yhdE::cat mreB* ^*E122D*^	This study
RU1711	BW25113 ∆*rodZ::kan* ∆*yhdE::cat mreB* ^*E122D*^	This study
RU1714	BW25113 *sfgfp‐rodZ* ∆*yhdE::cat mreB* ^*R124L*^	This study
RU1719	BW25113 *sfgfp‐rmr* ∆*yhdE::cat mreB* ^*R124L*^	This study
RU1709	BW25113 ∆*rodZ::kan* ∆*yhdE::cat mreB* ^*R124L*^	This study
RU1608	BW25113 *sfgfp‐rodZ* ∆*yhdE::cat mreB* ^*R124S*^	This study
RU1612	BW25113 *sfgfp‐rmr* ∆*yhdE::cat mreB* ^*R124S*^	This study
RU1598	BW25113 ∆*rodZ::kan* ∆*yhdE::cat mreB* ^*R124S*^	This study
RU1665	BW25113 *sfgfp‐rodZ* ∆*yhdE::cat mreB* ^*A125V*^	This study
RU1666	BW25113 *sfgfp‐rmr* ∆*yhdE::cat mreB* ^*A125V*^	This study
RU1597	BW25113 ∆*rodZ::kan* ∆*yhdE::cat mreB* ^*A125V*^	This study
RU1609	BW25113 *sfgfp‐rodZ* ∆*yhdE::cat mreB* ^*E137G*^	This study
RU1613	BW25113 *sfgfp‐rmr* ∆*yhdE::cat mreB* ^*E137G*^	This study
RU1605	BW25113 ∆*rodZ::kan* ∆*yhdE::cat mreB* ^*E137G*^	This study
RU1610	BW25113 *sfgfp‐rodZ* ∆*yhdE::cat mreC* ^*S153I*^	This study
RU1614	BW25113 *sfgfp‐rmr* ∆*yhdE::cat mreC* ^*S153I*^	This study
RU1606	BW25113 ∆*rodZ::kan* ∆*yhdE::cat mreC* ^*S153I*^	This study
RU1611	BW25113 *sfgfp‐rodZ* ∆*yhdE::cat mreD* ^*F123L*^	This study
RU1615	BW25113 *sfgfp‐rmr* ∆*yhdE::cat mreD* ^*F123L*^	This study
RU1607	BW25113 ∆*rodZ::kan* ∆*yhdE::cat mreD* ^*F123L*^	This study
DS673	BW25113 ∆*rlpA::cat*	Shiomi et al. (2013)
DS674	BW25113 ∆*rodZ::kan* ∆*rlpA::cat*	Shiomi et al. (2013)
RU1723	BW25113 ∆*rlpA::cat mrdA* ^*T52I*^	This study
RU1601	BW25113 *sfgfp‐rodZ* ∆*rlpA::cat mrdA* ^*T52I*^	This study
RU1616	BW25113 *sfgfp‐rmr* ∆*rlpA::cat mrdA* ^*T52I*^	This study
RU1599	BW25113 ∆*rodZ::kan* ∆*rlpA::cat mrdA* ^*T52I*^	This study
RU1670	BW25113 *sfgfp‐rodZ* ∆*rlpA::cat mrdA* ^*I59S*^	This study
RU1671	BW25113 *sfgfp‐rmr* ∆*rlpA::cat mrdA* ^*I59S*^	This study
RU1600	BW25113 ∆*rodZ::kan* ∆*rlpA::cat mrdA* ^*I59S*^	This study
RU1717	BW25113 *sfgfp‐rodZ* ∆*rlpA::cat mrdA* ^*A201V*^	This study
RU1722	BW25113 *sfgfp‐rmr* ∆*rlpA::cat mrdA* ^*A201V*^	This study
RU1712	BW25113 ∆*rodZ::kan* ∆*rlpA::cat mrdA* ^*A201V*^	This study
RU1715	BW25113 *sfgfp‐rodZ* ∆*rlpA::cat mrdA* ^*V227L*^	This study
RU1720	BW25113 *sfgfp‐rmr* ∆*rlpA::cat mrdA* ^*V227L*^	This study
RU1710	BW25113 ∆*rodZ::kan* ∆*rlpA::cat mrdA* ^*V227L*^	This study
RU1668	BW25113 *sfgfp‐rodZ* ∆*rlpA::cat mrdA* ^*R234L*^	This study
RU1669	BW25113 *sfgfp‐rmr* ∆*rlpA::cat mrdA* ^*R234L*^	This study
RU1667	BW25113 ∆*rodZ::kan* ∆*rlpA::cat mrdA* ^*R234L*^	This study
RU1713	BW25113 *sfgfp‐rodZ* ∆*rlpA::cat mrdB* ^*A234T*^	This study
RU1718	BW25113 *sfgfp‐rmr* ∆*rlpA::cat mrdB* ^*A234T*^	This study
RU1708	BW25113 ∆*rodZ::kan* ∆*rlpA::cat mrdB* ^*A234T*^	This study
RU1626	BW25113 *sfgfp‐rodZ* ∆*rlpA::cat mrdB* ^*K243N*^	This study
RU1627	BW25113 *sfgfp‐rmr* ∆*rlpA::cat mrdB* ^*K243N*^	This study
RU1625	BW25113 ∆*rodZ::kan* ∆*rlpA::cat mrdB* ^*K243N*^	This study
DHM1	∆*cyaA* strain for the BACTH assay	Karimova et al. (1998)

### 2.2 Strain construction

The primers used for the strain constructions are listed in Table 2. DNA polymerase Phusion or Taq (New England Biolabs) was used for polymerase chain reaction (PCR). Cells producing *sfGFP‐rmr* were constructed as follows: genomic DNA from RU382 (Ikebe et al., 2018) was amplified using primers 1266/841 and 842/18. The second PCR was carried out with these PCR products as templates and primers 1266 and 18. The PCR product was introduced into strain BW25113 carrying pKD46 (Datsenko & Wanner, 2000) by electroporation. Cells were selected on L plates containing 10 µg mL^−1^ Cm. The resulting strain was transformed with plasmid pCP20 by selection for ampicillin resistance (Amp^R^) at 30°C. The strain was then incubated at 42°C in the absence of Amp, and colonies that grew were screened for Amp^S^ and Cm^S^ phenotypes at 37°C. The resulting strain was designated as RU1353. A P1 lysate prepared from DS1317 (Kawazura et al., 2017) was used to transduce *mreB‐mCherry* ^*SW*^ *∆yhdE::cat* into RU1353 to yield RU1354 (*sfGFP‐rmr mreB‐mCherry* ^*SW*^). To transfer suppressor mutations, we performed the procedure as previously described (Shiomi et al., 2013). pKD3 was used as a template and primers 113 and 114 (for *yhdE*) or primers 287 and 288 (for *rlpA*) were used for PCR. PCR fragments containing a cat cassette flanked by an FLP recognition target site were inserted between the first and second codons of chromosomal *yhdE* (for suppressors in *mreB*, *mreC*, or *mreD*) or *rlpA* (for suppressors in *mrdA* or *mrdB*) genes in each suppressor strain carrying the λ Red expression plasmid pKD46 (Datsenko & Wanner, 2000). To transfer *mreB*, *mreC*, *mreD*, *mrdA*, and *mrdB* mutations, chloramphenicol‐resistant (Cm^R^) colonies were isolated after transformation of suppressors, which have mutations in *mreB*, *mreC*, *mreD*, *mrdA*, or *mrdB*, with PCR fragments to insert a cat resistance cassette in the *yhdE* gene. This gene is downstream of *mreD* (for *mreB*, *mreC*, and *mreD* mutations). A cat resistance cassette was also inserted into the *rlpA* gene, which is downstream of *mrdB* (for *mrdA* and *mrdB* mutations). This yielded RU1701 (*mreB* ^*E122D*^∆*yhdE*::cat), RU1647(*mreB* ^*R124L*^ ∆*yhdE*::cat), RU1640 (*mreB* ^*E137G*^∆*yhdE*::cat), RU1641 (*mreC* ^*S153I*^ ∆*yhdE*::cat), RU1642 (*mreD* ^*F123L*^ ∆*yhdE*::cat), RU1644 (*mrdA* ^*T52I*^ ∆*rlpA*::cat), RU1645 (*mrdA* ^*I59S*^ ∆*rlpA*::cat), RU1702 (*mrdA* ^*A201V*^ ∆*rlpA*::cat), RU1648 (*mrdA* ^*V227L*^ ∆*rlpA*::cat), RU1643 (*mrdA* ^*R234L*^ ∆*rlpA*::cat), and RU1646 (*mrdB* ^*K243N*^ ∆*rlpA*::cat). P1 phage was grown on a donor carrying *mreB*, *mreC*, or *mreD* mutations, and the *yhdE* gene was inserted with a cat resistance cassette, or *mrdA* or *mrdB* mutations, and the *rlpA* gene was inserted with a cat resistance cassette, and were used to transduce RU383 (sfGFP‐RodZ), RU1353 (sfGFP‐RMR), or RU2 (∆*rodZ::kan*). Fresh transductants were restreaked on L plates containing Cm, and Cm^R^ clones were selected. All the mutation sites were sequenced and confirmed. The resultant strains are listed in Table 1 and were used for further analyses.

**Table 2:** **Primers used in this study**.

Primer (number)	Sequence
*ispG*‐r41 (18)	CGGCACATTCCCAACGTAAATAC
RMTM1R‐r (841)	TTCCCCTTGTGCGTACATTAAAACAACAAGGTAACCCACCAGCAGGCCGAGCAGACCTAGCACTGACCAGCCGTCGCGTTTTTTGCGGC
RMR‐f‐1 (842)	GCTGCCAGGGCTGGAAATGGATGTCATTAAAAAG
*sfgfp‐rodZ*1‐2 (1266)	GATGGTTCACCGGCATCTCAATTCTCATTTAAACGTACCTGCAGCGAATGGTGGAGGCTGGAGCTGCTTC
*yhdE*‐H1P1 (113)	GCGCAAAGTCCGTCAGCAGTTTGCAGTGCAATAAAGGTTTCTATGGTGTAGGCTGGAGCTGCTTC
*yhdE*‐H2P2 (114)	GTAACTCCTGACGACGCGGAGAACCGGAAGCTAAATACAGAGAAGTCATATGAATATCCTCCTTA
*rlpA*‐f H1P1 (287)	CAGGAAAATGTTGTCGAAAAGCGTGTAAGAGGTGCGCAATGGTGTAGGCTGGAGCTGCTTC
*rlpA*‐r H2P2 (288)	CGAGCATTCCTGCCGCGATGCAGATCCCGAGCCACTGCTTCATATGAATATCCTCCTTA
*yfgA*‐f (BamHI) for B2H (35)	GCGGATCCCAATACTGAAGCCACGCACG
*yfgA*‐r (EcoRI) (36)	GCGAATTCTTACTGCGCCGGTGATTG
PBP2‐f (BamHI) (824)	GCGGATCCCAAACTACAGAACTCTTTTCG
PBP2‐r (EcoRI) (825)	GCGAATTCTAATGGTCCTCCGCTGCGGC
RodA‐f (BamHI) (830)	GCGGATCCCACGGATAATCCGAATAAAAAAAC
RodA‐r (EcoRI) (831)	GCGAATTCTTACACGCTTTTCGACAAC

### 2.3 Plasmid constructions for the BACTH assay

Primers and plasmids used in this study are listed in Tables 2 and 3, respectively. *rmr*, *mrdA*, *mrdB*, *mrdB‐A234T*, and *mrdB‐K243N* were amplified using RU1353 (*sfGFP‐rmr*), BW25113 (WT for *mrdA* and *mrdB*), DS686 (*mrdB‐A234T*), and RU1646 (*mrdB‐K243N*) and primers 35/36 for *rmr*, 824/825 for *mrdA*, and 830/831 for *mrdB* and its mutants. The PCR products were cut with BamHI and EcoRI, and the fragments were cloned into the corresponding site of pKT25 or pUT18C to yield pDS1274 (rmr in pKT25), pDS1353 (mrdA in pKT25), pDS1351 (mrdB in pKT25), pDS1352 (mrdB in pUT18C), pRU1622 (*mrdB‐A234T* in pUT18), and pRU1899 (*mrdB‐K243N* in pUT18).

**Table 3:** **Plasmids used in this study**.

Plasmid	Relevant genotype	Reference
pKT25	P_*lac*_::T25, Kan^R^	Karimova et al. (1998)
pUT18C	P_*lac*_::T18, Amp^R^	Karimova et al. (1998)
pDS1271	*rodZ* in pKT25, Kan^R^	Yoshii et al. (2019)
pDS1274	*rmr* in pKT25, Kan^R^	This study
pDS1266	*rodZ* in pUT18C, Amp^R^	Yoshii et al. (2019)
pDS1269	*rmr* in pUT18C, Amp^R^	Yoshii et al. (2019)
pTK554	*mreB* in pKT25, Kan^R^	Kruse et al. (2005)
pRU1059	*mreC* in pUT18C, Amp^R^	Kruse et al. (2005)
pRU1077	*mreD* in pUT18C, Amp^R^	Kruse et al. (2005)
pDS1353	*mrdA* in pKT25, Kan^R^	This study
pDS1351	*mrdB* in pKT25, Kan^R^	This study
pDS1352	*mrdB* in pUT18C, Amp^R^	This study
pRU1622	*mrdB‐A234T* in pUT18C, Amp^R^	This study
pRU1899	*mrdB‐K243N* in pUT18C, Amp^R^	This study
pKD3	*FRT‐cat‐FRT*, Cm^*R*^ Amp^R^	Datsenko and Wanner (2000)
pKD46	Lambda Red recombinase, Amp^R^	Datsenko and Wanner (2000)
pCP20	yeast Flp recombinase gene, Cm^R^	Datsenko and Wanner (2000)

### 2.4 Microscopic observations

Cells were grown in L medium to log phase at 37°C (unless otherwise stated) and mounted on 2% agarose in M9 medium (0.6% Na_2_HPO_4_, 0.3% K_2_HPO_4_, 0.05% NaCl, 0.1% NH_4_Cl, 0.1 mM MgSO_4_·7H_2_O, 0.2% glucose) (M9‐agarose pad). Cells were observed using an Axio Observer (Zeiss), and images were processed using ZEN (Zeiss), Photoshop 2020 (Adobe), and ImageJ. All experiments were repeated two or more times on different days.

### 2.5 Image analyses

Cells were detected and counted automatically using ImageJ and its plug‐in MicrobeJ (Ducret et al., 2016) or EzColocalization (Stauffer et al., 2018). All but overlapping cells in the images were counted.

### 2.6 Isolation of suppressors of the slow‐growth phenotype of RMR cells

Isolation of suppressors of the slow‐growth phenotype of RU1353 (sfGFP‐RMR) was performed as previously described (Shiomi et al., 2013). Briefly, several different colonies of strain RU1353 were cultured in L medium at 37°C, diluted 100‐fold in new L medium the next day, and cultured further at 37°C. After repeating this inoculation for 1 week, the bacterial cells were spread on L‐plates. Large and small colonies appeared, and large colonies were isolated as suppressors.

### 2.7 Whole‐genome sequencing and SNP genotyping

Genomic DNA was purified from each suppressor strain using the Wizard Genomic DNA Purification Kit (Promega). One microgram of genomic DNA was sheared using an M220 focused ultrasonicator (Covaris) to obtain peak fragment lengths of 500–600 bp. Next, the NEBNext Ultra DNA Library Preparation kit (New England Biolabs) was used to repair the ends and to add the Illumina MiSeq‐compatible barcode adapters to fragmented DNA. The resulting fragments were size‐selected using Agencourt AMPure XP bead sizing (Beckman Coulter). Indexes were then added in a limited‐cycle PCR (7 cycles), followed by purification on Agencourt AMpure XP beads. After the 2 × 250 bp Illumina MiSeq paired‐end sequencing run, the data were base‐called, and reads with the same barcode were collected and assigned to a sample on the instrument, which generated Illumina FASTQ files. Mapping and SNP detection were performed using the BWA (Li & Durbin, 2009) in the DDBJ Read Annotation Pipeline (Nagasaki et al., 2013). The genome sequence of MG1655 (Accession number: NC_000913) was used as the reference sequence for genome mapping.

### 2.8 Purification of peptidoglycan and observation of the structure of peptidoglycan revealed by a QFDE‐EM

Cells grown overnight at 37°C in L medium were diluted 100‐fold with fresh L medium (200 mL) and further grown at 37°C to late‐log phase (OD_600_ = approx 1.0). The cells were centrifuged for 5 min. Next, the pellet was washed with 4 mL of distilled water, centrifuged at 4400*g* for 5 min, and the precipitate was suspended in 8 mL of 10% SDS and heated at 100°C for 20 h. Thereafter, the precipitate obtained by centrifugation at 20,000*g* for 30 min was washed with 6 mL of distilled water and centrifuged at 20,000*g* for 30 min. The precipitate was suspended in 200 μL of PBS and treated with 300 μg/mL trypsin at 37°C for 22 h. The precipitate obtained by centrifugation at 20,000*g* for 30 min was suspended in 250 µL of 1% SDS and heated at 100°C for 2 h. Thereafter, the precipitate obtained by centrifugation at 20,000*g* for 30 min was washed with 500 μL of distilled water and centrifuged at 20,000*g* for 30 min. Finally, the pellet was suspended in 200 μL of distilled water to obtain a purified PG sample. Purified peptidoglycan was observed using QFDE‐EM. Observations were performed as previously described (Tulum et al., 2019). Pores smaller than 4 nm^2^ and larger than 1000 nm^2^ were excluded from the quantification analysis.

### 2.9 Sacculus composition analysis

Peptidoglycan compositions in *E. coli* strains were analyzed as described previously (Desmarais et al., 2014; Kühner et al., 2014). Cells were cultivated in 250 mL of LB medium until OD_600_ = 0.7–0.8 and harvested by centrifugation at 5000*g* for 10 min. Harvested cells were immediately resuspended to 3 mL of LB medium and dropped into 6 mL of boiled 6% SDS with stirring to lyse the cells (the final concentration of the SDS solution was 4% SDS). The cells were continuously boiled for 3 h and cooled down at room temperature with stirring overnight. To remove SDS completely from Sacculi, which were repeatedly washed with water and ultracentrifuged (himac CS150XG2; Hitachi) at 45,000*g* for 40 min at room temperature. SDS‐free sacculi were resuspended in 900 µL of 10 mM Tris‐HCl (pH 7.2) with 0.06% (w/v) NaCl and treated with 100 µg/mL Pronase E (Merck), at 60°C for 2 h. The Pronase E digestion was stopped by adding 200 µL of 6% SDS to the sample and incubating samples at 100°C for 30 min. The samples were repeatedly washed with water and ultracentrifuged to remove SDS completely again. The samples were resuspended in 200 µL of 50 mM sodium phosphate buffer (pH 4.9), treated with 40 µg/mL muramidase (Sigma‐Aldrich), and incubated at 37°C overnight. Muramidase digestion was stopped by incubating samples at 100°C for 5 min. To prepare the sample for the LC/MS analysis, the sample was centrifuged at 16,000*g* for 10 min at room temperature and the supernatant was collected. To adjust pH of the sample, an adequate volume of 500 mM boric acid solution was added to the sample (the final concentration was 100 mM boric acid). The sample was then added 8 ~ 10 grains of sodium borohydride (Tokyo Chemical Industry). The sample was finally added 50% (v/v) orthophosphoric acid (Sigma‐Aldrich) to adjust the final pH of the sample to pH 3.0–4.0. The sample was stored at −80°C until the LC/MS analysis. The sample was diluted by one‐third with water before being applied for LC/MS analysis. LC/MS was performed using Acquity UPLC H‐Class PLUS (Waters) and MALDI Synapt G2‐Si HDMS (Waters) coupled with electrospray ionization (ESI) source. Data acquisition and processing were performed using MassLynx 4.2. The sample injection volume was 5 µL, and the column temperature was 40°C. An Inertsil ODS‐HL column (1.9 μm 2.1 × 100 mm, GL Sciences) was used at a flow rate of 0.176 mL/min. The mobile phase consisted of A (water containing 0.1% formic acid) and B (acetonitrile containing 0.1% formic acid). The gradient program occurred for 75 min as follows: 0 min to 5% B, 1 min to 5% B, 60 min to 30% B, 60.1 min to 98% B, 65 min to 98% B, 65.1 min to 5% B, and 75 min to 100% B. The flow between 0 and 5 min was systematically diverted to the waste using a switching valve. The ESI capillary voltage was set to 2.0 kV, and the sampling cone voltage was 100 V. Source and desolvation temperatures were 125°C and 450°C, respectively. The desolvation gas flow was set at 800 L/h. Injection voltages into the trap and transfer cells were 4 and 2 V, respectively. Argon gas flowed into the trap and the transferred cells. The structural assignments of the detected peptides were determined based on both the accurate mass measured in the LC/MS experiment and the previous report (Kühner et al., 2014). The peak areas of individual ions were normalized to the area of unidentified ions (RT 10.3) in each LC/MS chromatogram, which were detected in all experiments constantly.

## 3 RESULTS

### 3.1 Characterization of cells producing chimeric protein RMR

**Figure A1:**
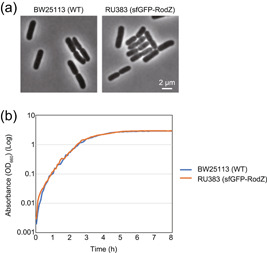
Comparison between BW25113 (WT) and RU383 (sfGFP‐RodZ). Phase contrast images (a) and growth curve (b) of BW25113 and RU383.

To investigate the relationship between the function and localization of the Rod complex and the structure of peptidoglycan, we analyzed mutant strains of the Rod complex. Most of the Rod complex components, such as MreB and PBP2, are essential for cell growth, but RodZ is nonessential in L medium. However, the morphology of the ∆*rodZ* strain differs greatly from that of the wild‐type strain, as it is spherical or oval (Bendezú et al., 2009; Shiomi et al., 2008). Previously, we constructed a chimeric protein, called RMR, in which the transmembrane domain of RodZ (RodZ^112‐133^) was replaced with the first transmembrane domain of MalF (MalF^17‐39^) (Figure 1b) (Yoshii et al., 2019). MalF protein is not directly involved in peptidoglycan synthesis, and chimeric proteins of MalF with various proteins have been constructed and analyzed (Guzman et al., 1997). The protein amount of the RMR protein was the same as that of RodZ, indicating that the stability of the RodZ protein was not compromised by this replacement (Yoshii et al., 2019). Morphological abnormalities of cells producing RMR were found, but they were not as severe as those in ∆*rodZ* cells (Figure 1b,c). Therefore, we analyzed the morphology, growth rate, and subcellular localization of RMR‐producing cells. Here, we compared cells producing sfGFP‐RodZ with cells producing sfGFP‐RMR and with ∆*rodZ* cells. Before the analyses of cells producing sfGFP (super‐folder green fluorescent protein) tagged RodZ or RMR, we investigated the morphology and growth rate of cells producing RodZ or sfGFP‐RodZ (Figure A1a,b) in which sfGFP was fused with the N‐terminus of RodZ (Ikebe et al., 2018). The average cell length (L) and width (W) ± standard deviation of cells producing RodZ or sfGFP‐RodZ were 4.19 ± 0.91 µm (L) and 0.93 ± 0.08 µm (W) (RodZ) and 3.25 ± 0.75 µm (L) and 0.94 ± 0.04 µm (W) (sfGFP‐RodZ), indicating that sfGFP slightly affected length but not width. The doubling time of both cells producing RodZ or sfGFP‐RodZ was 30 min (Figure A1b and Table 4). Thus, we concluded that the fusion of sfGFP with RodZ does not have much of a negative effect on RodZ function.

**Table 4:** Growth rate, length, and width of cells carrying a suppressor mutation.

Mutation	*rmr* (sfGFP‐RMR)			*rodZ* (sfGFP‐RodZ)			*∆rodZ*		
	Growth rate (min)	Length^a^ (µm)	Width^a^ (µm)	Growth rate (min)	Length (µm)	Width (µm)	Growth rate (min)	Length (µm)	Width (µm)
	36	2.68 ± 0.72	1.40 ± 0.24	30	3.25 ± 0.75	0.94 ± 0.04	48	2.35 ± 0.69	1.56 ± 0.27
*mreB* ^*E122D*^ d	31	3.45 ± 0.83^b^, ^d^	1.05 ± 0.09^b^, ^d^	30	3.36 ± 1.07^b^	0.91 ± 0.13^b^	33	2.80 ± 0.79^b^, ^f^	1.20 ± 0.27^b^, ^f^
*mreB* ^*R124L*^	31	3.24 ± 0.84^c^, ^d^	0.96 ± 0.07^b^, ^d^	30	4.21 ± 1.26^b^	0.96 ± 0.08^b^	31	3.07 ± 0.86^b^, ^f^	1.08 ± 0.11^b^, ^f^
*mreB* ^*R124S*^	28	3.19 ± 1.14^c^, ^d^	1.04 ± 0.13^b^, ^d^	28	4.16 ± 2.39^b^	0.83 ± 0.09^b^	31	2.73 ± 0.80^b^, ^f^	1.13 ± 0.28^b^, ^f^
*mreB* ^*A125V*^	30	3.85 ± 1.06^b^, ^d^	0.96 ± 0.06^b^, ^d^	27	4.39 ± 2.11^b^	0.87 ± 0.07^b^	31	3.38 ± 0.97^b^, ^f^	1.08 ± 0.16^b^, ^f^
*mreB* ^*E137G*^	28	2.71 ± 0.72^b^, ^e^	1.10 ± 0.08^b^, ^d^	28	2.97 ± 0.74^b^	0.91 ± 0.05^b^	30	2.51 ± 0.66^b^, ^f^	1.25 ± 0.12^b^, ^f^
*mreC* ^*S153I*^	30	2.91 ± 0.73^b^, ^d^	1.12 ± 0.11^b^, ^d^	29	3.29 ± 0.87^c^	0.90 ± 0.05^b^	33	2.78 ± 0.70^b^, ^f^	1.35 ± 0.19^b^, ^f^
*mreD* ^*F123L*^	29	2.83 ± 0.69^b^, ^d^	0.93 ± 0.06^b^, ^d^	29	3.31 ± 0.99^c^	0.86 ± 0.05^b^	28	2.41 ± 0.65^b^, ^f^	1.07 ± 0.08^b^, ^f^
*mrdA* ^*T52I*^	30	3.27 ± 0.81^c^, ^d^	1.16 ± 0.19^b^, ^d^	28	3.44 ± 1.32^b^	0.85 ± 0.05^b^	31	2.94 ± 0.78^b^, ^f^	1.28 ± 0.20^b^, ^f^
*mrdA* ^*I59S*^	31	3.35 ± 0.92^c^, ^d^	1.18 ± 0.26^b^, ^d^	30	5.62 ± 3.96^b^	0.80 ± 0.06^b^	31	2.83 ± 0.81^b^, ^f^	1.26 ± 0.28^b^, ^f^
*mrdA* ^*A201V*^	32	3.48 ± 0.98^b^, ^d^	1.35 ± 0.20^b^, ^d^	31	2.83 ± 0.68^b^	0.95 ± 0.07^b^	35	2.51 ± 0.65^b^, ^f^	1.53 ± 0.24^b^, ^g^
*mrdA* ^*V227L*^	32	3.18 ± 0.94^c^, ^d^	1.12 ± 0.20^b^, ^d^	32	4.39 ± 2.34^b^	0.83 ± 0.05^b^	32	2.82 ± 0.72^b^, ^f^	1.17 ± 0.19^b^, ^f^
*mrdA* ^*R234L*^	30	2.96 ± 0.80^b^, ^d^	1.17 ± 0.10^b^, ^d^	29	3.17 ± 0.74^c^	0.95 ± 0.05^c^	31	3.12 ± 0.90^b^, ^f^	1.43 ± 0.20^b^, ^f^
*mrdB* ^*A234T*^	32	3.41 ± 0.86^b^, ^d^	1.02 ± 0.13^b^, ^d^	30	3.67 ± 1.44^b^	0.79 ± 0.05^b^	32	2.41 ± 0.63^b^, ^g^	1.09 ± 0.13^b^, ^f^
*mrdB* ^*K243N*^	30	3.12 ± 0.80^b^, ^d^	1.14 ± 0.17^b^, ^d^	28	3.39 ± 1.06^b^	0.85 ± 0.04^b^	32	2.79 ± 0.76^b^, ^f^	1.25 ± 0.18^b^, ^f^

^a^ Mean length and width ± standard deviation (SD) are shown.

^b^ Significantly different from length or width of RU383 (*p* value < 0.05). *p* Values were determined by unpaired *T* test.

^c^ Not significantly different from length or width of RU383 (*p* value > 0.05). *p* Values were determined by unpaired *T* test.

^d^ Significantly different from length or width of RU1353 (*p* value < 0.05). *p* Values were determined by unpaired *T* test.

^e^ Not significantly different from length or width of RU1353 (*p* value > 0.05). *p* Values were determined by unpaired *T* test.

^f^ Significantly different from length or width of RU2 (*p* value < 0.05). *p* Values were determined by unpaired *T* test.

^g^ Not significantly different from length or width of RU2 (*p* value > 0.05). *p* Values were determined by unpaired *T* test.

We then calculated the growth rate of cells producing sfGFP‐RodZ (hereafter simply referred to as RodZ or WT), sfGFP‐RMR (hereafter simply referred to as RMR), and cells lacking *rodZ* as a control. We found that the doubling times of cells producing RodZ and RMR or cells lacking *rodZ* were 30 min (WT), 36 min (RMR), and 48 min (∆*rodZ*) (Table 4). Then, we examined the shape of cells producing RMR and ∆*rodZ* cells as a control (Figure 1 and Table 4). Cells producing RodZ (WT) showed a rod shape, while cells producing RMR or cells lacking *rodZ* showed a round or oval shape. We measured the cell length and width (Figure 1a–c, and Table 4). The average length (L) and width (W) ± standard deviation were 2.68 ± 0.72 µm (L) and 1.40 ± 0.24 µm (W) (RMR), and 2.35 ± 0.69 µm (L) and 1.56 ± 0.27 µm (W) (∆*rodZ*). As described above, the average cell length (L) and width (W) ± standard deviation of cells producing RodZ were 3.25 ± 0.75 µm (L) and 0.94 ± 0.04 µm (W). These results indicate that RMR did not completely lose the function of RodZ, and was an intermediate phenotype between WT and ∆*rodZ*.

### 3.2 Cluster formations of RMR

**Figure 2:**
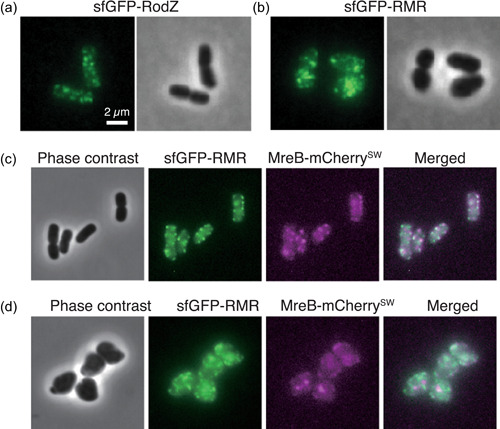
Subcellular localization of Rod complex. Subcellular localization of sfGFP‐RodZ (a) and sfGFP‐RMR (b). Subcellular localization of sfGFP‐RodZ and MreB‐mCherry^SW^ (c) and sfGFP‐RMR and MreB‐mCherry^SW^ (d) in a single cell. Phase contrast and fluorescent images are shown.

**Figure A2:**
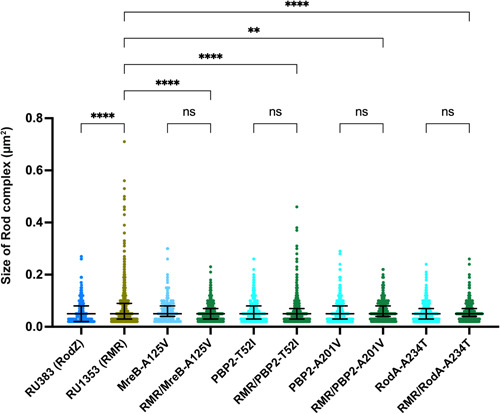
Size of the Rod complex. Violin plots showing the distribution of the size of the Rod complex estimated from images of sfGFP‐RodZ or sfGFP‐RMR in the indicated strains. Average and standard deviation (S.D.) are shown. *p* Values were determined by unpaired *T* test. ns: *p* > 0.05, *p ≤ 0.05, **p ≤ 0.01, ***p ≤ 0.001, ****p ≤ 0.0001.

**Figure A3:**
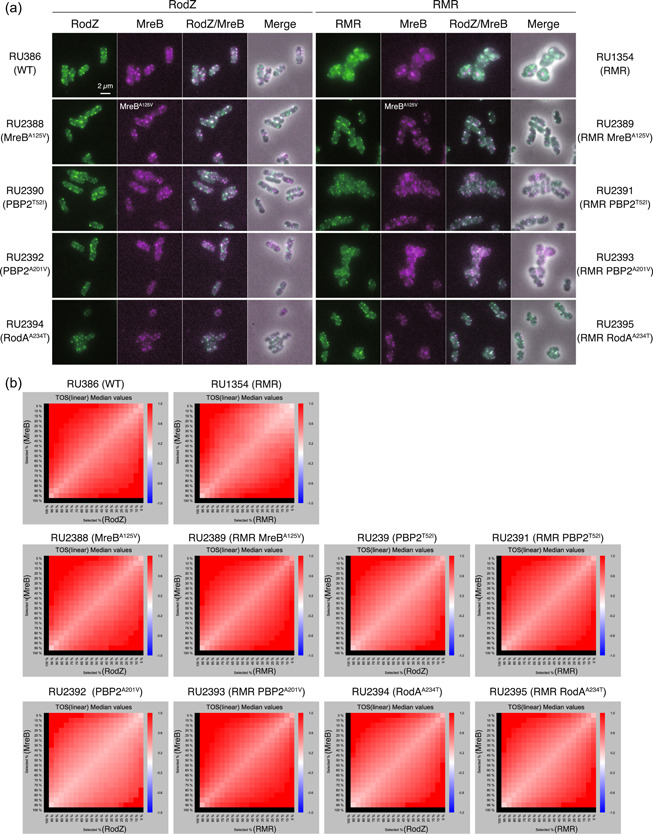
Colocalization of MreB and RodZ or RMR in cells producing each suppressor. (a) Cells producing MreB‐mCherry^SW^ and sfGFP‐RodZ or sfGFP‐RMR were grown to log‐phase in L medium at 37°C. Fluorescent and merged images are shown. (b) Quantitative analyses of colocalization of MreB‐mCherry^SW^ and sfGFP‐RodZ or sfGFP‐RMR. Metric matrixes for the threshold overlap score (TOS) (linear scaling) for each strain are shown.

**Figure A4:**
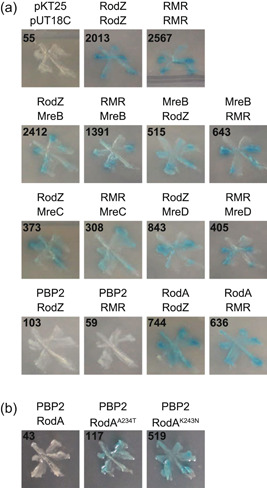
Interactions in the Rod complex. Interactions were examined by the bacterial two‐hybrid assay. DHM1 cells carrying plasmids producing the indicated proteins fused to either the T18 or T25 fragment of adenylate cyclase were streaked on a plate containing 5‐Bromo‐4‐Chloro‐3‐Indolyl‐β‐d‐Galactoside (X‐gal). The plates were incubated at 37°C for overnight. The number in each picture shows the average Millar units of two independent experiments. (a) Self‐interactions of RodZ and RMR and interactions between RodZ (RMR) and each component of the Rod complex are shown. (b) Interactions between PBP2 and RodA or its mutants are shown.

Next, we observed the subcellular localization of RodZ and RMR using epifluorescence microscopy. To observe sfGFP‐RodZ and sfGFP‐RMR, which are transmembrane proteins, we attempted to image fluorescence at the cell surface. Therefore, the phase contrast images that were simultaneously captured were slightly out‐of‐focus (Figure 2b). sfGFP‐RodZ formed clusters in the cylindrical part of the cell, as previously shown (Shiomi et al., 2008; Yoshii et al., 2019) (Figure 2a). sfGFP‐RMR also formed clusters, and some clusters were very bright as if they were aggregated in the cell (Figure 2b). We also observed very faint fluorescence of sfGFP‐RMR throughout the cell surface (Figure 2b). To more closely observe the Rod complexes, we simultaneously observed sfGFP‐RodZ/sfGFP‐RMR and MreB‐mCherry^SW^. WT RodZ and MreB were colocalized, as previously observed (Alyahya et al., 2009; Bendezú et al., 2009; Shiomi et al., 2008) (Figure 2c). Some clusters of RMR and MreB were brighter/larger or darker than others (Figure 2d). Furthermore, the darker clusters were relatively scattered throughout the cell surface. Localization of the Rod complex was then quantified by measuring the area on the image of fluorescence emitted from sfGFP‐RodZ or sfGFP‐RMR (Figure A2). We found that the Rod complex containing RMR was significantly larger than that containing RodZ. These results suggest that the Rod complex containing RMR is somewhat different from that containing WT RodZ. Image analysis revealed that most RodZ or RMR colocalized with MreB, but the degree of colocalization was somewhat reduced for RMR compared with RodZ (Figure 2c,d; Figure A3a,b). These results suggest that some RMR failed to form proper clusters, unlike WT RodZ, and may have led to cells producing RMR showing an abnormal shape and slow growth phenotype. It is possible that RMR lost abilities to interact with itself and other proteins so RMR failed to form proper clusters. Thus, we examined those interactions by the bacterial‐two hybrid assay (BACTH assay) (Karimova et al., 1998). RMR retained abilities to interact with itself and other proteins although we could not detect the interaction between RodZ/RMR and PBP2 (Figure A4a). This result suggests that RMRcano interacts with each protein but would not be able to organize the overall structure of the Rod complex. The transmembrane domain of RodZ likely plays a role in forming or stabilizing the proper structure of the Rod complex.

### 3.3 Isolation of mutants suppressing the slow‐growth phenotype of the *rmr* cells

**Figure 3:**
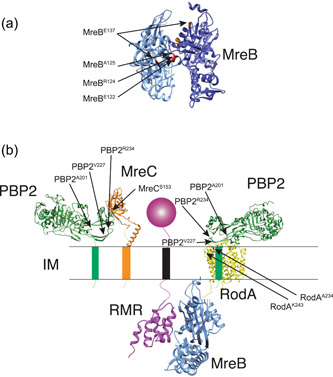
Mapping of the suppressor mutations on 3D structural models of *E. coli* Rod complex. Suppressor mutations isolated in MreB (a) and other proteins (b) are shown.

Previously, we isolated suppressor mutations of the slow‐growth phenotype of ∆*rodZ* cells (Shiomi & Niki, 2013; Shiomi et al., 2013). Most of the suppressor mutations were found in the components of the Rod complex. We expected that if we isolated suppressors of RMR cells, we would find mutations in the interaction sites between RodZ and other proteins, in addition to mutations in the components of the Rod complex. To isolate the suppressor mutants of RMR cells, several independent colonies of RU1353 (*sfgfp‐rmr*) cells were grown in L medium at 37°C overnight. The cells were diluted in fresh L medium the next morning and grown the next day at 37°C, and this cultivation was repeated for 1 week. Then, the cells were plated on an L agar plate, and the plates were incubated at 37°C overnight. It is known that ∆*rodZ* cells which grow slower than WT cells form smaller colonies on the L agar plate (Shiomi et al., 2008, 2013). Thus, if larger colonies emerged, it would be suppressor mutants of the slow‐growth phenotype of RMR cells. Larger and smaller colonies emerged. We independently isolated 18 of these large‐colony suppressor mutants. We could determine the mutation sites in 16 out of 18 suppressors by whole‐genome sequencing. Some of the suppressor mutations isolated in this study had already been isolated in the previous study, in which we isolated suppressors of the slow‐growth phenotype of ∆*rodZ* mutant (Shiomi et al., 2013) (Table 5). Unexpectedly, no mutations were found in the RMR itself. Instead, all of the mutations (15 mutations), except for one mutation, were found in *mreB*, *mreC*, *mreD*, *mrdA* encoding PBP2, or *mrdB* encoding RodA, which are involved in the Rod complex. We will report the suppressor mutation occurring outside the Rod complex in a separate paper. Suppressor mutations were mapped onto a three‐dimensional structural model of each protein (Figure 3). Many of the suppressor mutations were located at the protein–protein interaction surfaces, suggesting that the suppressors could have altered the protein–protein interactions of the Rod complex components to compensate for RMR. Indeed, we previously showed that MreB^A125V^ exhibited stronger interactions with itself (self‐interaction) and MreC than WT MreB (Shiomi et al., 2013). It was shown that the PBP2^L61R^ mutant suppresses a MreC defect (Rohs et al., 2018) and activates the GTase activity of RodA without changing the interaction of RodA and PBP2 (Liu et al., 2020). Therefore, it is plausible to hypothesize that PBP2^T52I^ and PBP2^I59S^ mutants activate the GTase activity of RodA because Thr52 and Ile59 in PBP2 are close to Leu61. We also examined interactions between PBP2 and RodA^A234T^ or RodA^K243N^ by the BACTH assay and found that RodA^A234T^ and RodA^K243N^ showed stronger interaction with PBP2 than WT RodA although we could not detect interaction between PBP2 and WT RodA (Figure A4b). This would be consistent with the observation that RodA^A234T^ has a stronger activity to synthesize peptidoglycan (Rohs et al., 2018). Therefore, these mutations may increase the activity of the Rod complex.

**Table 5:** Suppressor mutations isolated in this study.

Amino acid mutation site	Replaced by	Base substitution
*mreB* ^*E122*^	Asp	A^366^ to C
*mreB^R124^* a	Ser	C^370^ to A
*mreB^R124^* b	Leu^c^	G^371^ to A
*mreB^A125^* a	Val	C^374^ to T
*mreB* ^*E137*^ b	Gly	A^410^ to G
*mreC* ^*S153*^	Ile	G^458^ to T
*mreD* ^*F123*^	Leu	T^367^ to C
*mrdA* ^*T52*^ b	Ile	C^155^ to T
*mrdA* ^*I59*^	Ser	T^176^ to G
*mrdA* ^*A201*^	Val	C^602^ to T
*mrdA* ^*V227*^	Leu	G^679^ to C
*mrdA* ^*R234*^	Leu	G^701^ to T
*mrdB^A234^* a	Thr	G^700^ to A
*mrdB* ^*K243*^	Asn	A^729^ to C

^a^ These mutations were isolated as suppressor mutations in ∆*rodZ* cells (Shiomi et al., 2013).

^b^ These amino acids were mutated in suppressors of ∆*rodZ*, but the amino acids replaced were different (Shiomi et al., 2013).

^c^ *mreB^R124L^* mutation was isolated in two independent suppressors.

**Figure A5:**
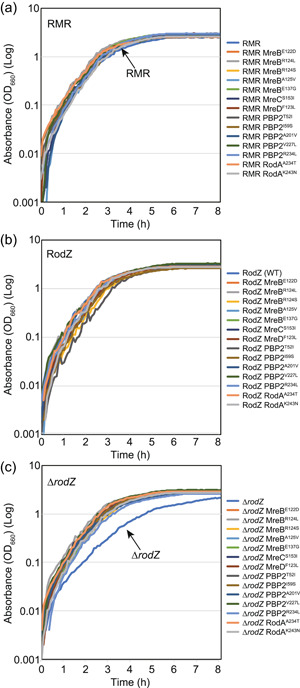
Growth of cells producing each suppressor. Cells were grown in L medium at 37°C and OD_660_ was automatically measured. Growth curves of RMR (a), RodZ (b), or ∆*rodZ* (c) cells producing each suppressor are shown.

Before we analyzed the suppressor mutants, we transferred all of the suppressor mutations (mutations in *mreB*, *mreC*, *mreD*, *mrdA*, or *mrdB*) to WT and RMR strains. We also transferred the mutations into ∆*rodZ* cells to examine whether the mutations were capable of restoring the slow‐growth phenotype of cells lacking *rodZ*. The growth rates were calculated (Table 4 and Figure A5). None of the suppressor mutations significantly affected the growth rate of the WT strain, but restored the slow‐growth phenotype of RMR and ∆*rodZ* cells (Table 4 and Figure A5), indicating that the mutations isolated as suppressors of the slow‐growth phenotype of RMR cells could also suppress the slow‐growth phenotype of ∆*rodZ* cells.

### 3.4 Characterizations of the suppressors

**Figure A6:**
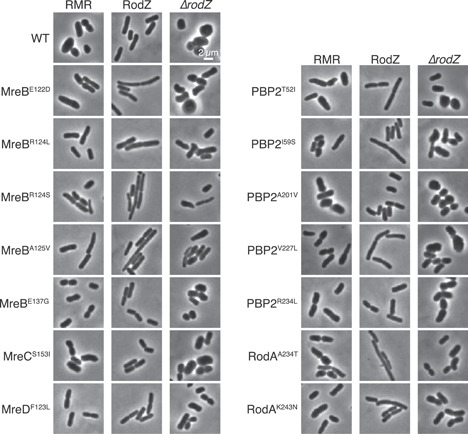
Cell morphology of cells producing each suppressor. RMR, RodZ, or ∆*rodZ* cells producing each suppressor were grown to log‐phase in L medium, and phase contrast images were taken.

When we previously isolated the suppressors of ∆*rodZ* cells, the suppressor mutations restored not only the growth rate but also the cell shape. Therefore, we next observed the cell shape and measured the length and width of all strains carrying suppressor mutations (Figure A6 and Table 4). We found that all the mutations completely or partially restored the rod shape, although the cell width of RMR and ∆*rodZ* cells was variable, with the distribution of the width of the suppressor strains producing RMR or lacking *rodZ* being relatively narrow.

**Figure 4:**
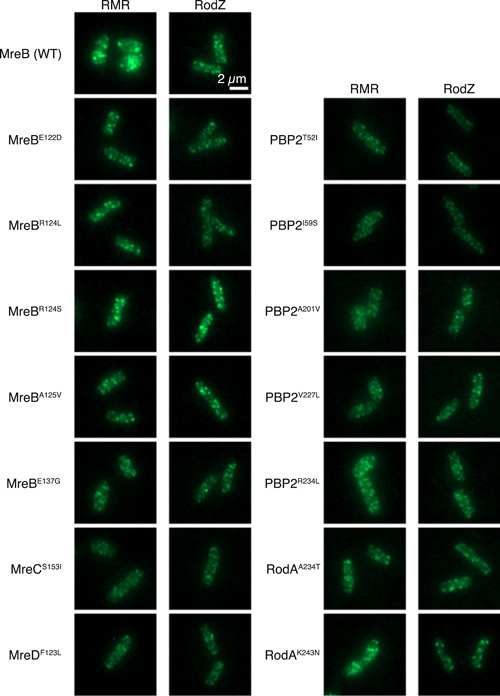
Subcellular localization of RodZ or RMR. Subcellular localization of sfGFP‐RodZ or sfGFP‐RMR in cells producing each suppressor. Typical cells are shown.

To investigate whether these suppressor mutations restored Rod cluster formation, we observed the subcellular localization of RodZ and RMR (Figure 4 and Figure A2) and colocalization with MreB in cells producing suppressors (Figure A3). As described above, sfGFP‐RMR formed brighter and larger clusters. However, sfGFP‐RMR in cells carrying suppressor mutations formed clusters similar to those of sfGFP‐RodZ. We also quantitatively analyzed the Rod complex formation (Figure A2) and colocalization between RodZ or RMR and MreB in the suppressors (Figure A3). These results suggest that the suppressor mutations restored the assembly of the Rod complex even though the strains had RMR in the Rod complex.

**Figure 5:**
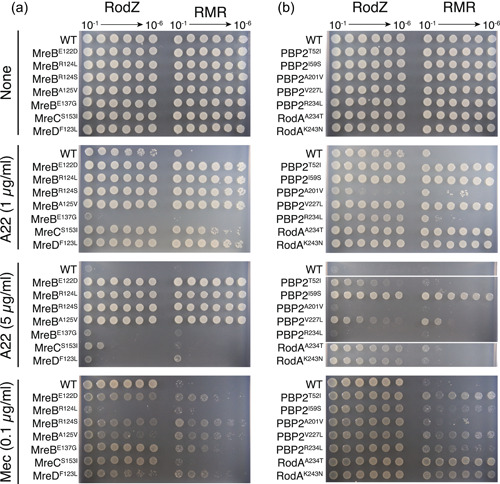
Sensitivity of cells producing suppressor mutations to antibiotics. Sensitivity of cells producing *mreB*, *mreC*, or *mreD* (a) or *mrdA* (encoding PBP2) or *mrdB* (encoding RodA) (b) to A22 and mecillinam. An overnight culture of the indicated strains was diluted serially (from 10^−1^ to 10^−6^) and spotted onto L plates containing A22 or mecillinam. The plates were incubated for 24 h at 37°C.

We previously showed (Shiomi et al., 2013) that ∆*rodZ* cells are more sensitive to the antibiotics A22 (Iwai et al., 2002), which inhibit the binding of ATP to MreB (Bean et al., 2009) and mecillinam, which inhibits the transpeptidase activity of PBP2 (Spratt, 1975). We found that RMR cells were also more sensitive to both antibiotics (Figure 5). If the suppressor mutations improved the assembly of the Rod complex, the cells would be resistant to these antibiotics. We examined the sensitivity to A22 of RodZ or RMR cells carrying suppressor mutations. As shown in Figure 5a, RodZ or RMR cells producing MreB^E122D^, MreB^R124L^, MreB^R124S^, or MreB^A125V^ were resistant to 5 µg/mL A22, whereas RodZ or RMR cells were not viable under the same conditions, supporting the idea that these mutations promote the assembly of MreB filaments, hence the Rod complex. Interestingly, RodZ or RMR cells producing MreB^E137G^ were more sensitive to 1 µg/mL A22 than RodZ or RMR cells. Because MreB^E137G^ is located closer to the cytoplasmic membrane, MreB^E137G^ must suppress the RMR phenotype differently than other MreB suppressor mutants, such as by increasing MreB membrane binding and/or interacting with other proteins. RMR cells producing MreC^S153I^ or MreD^F123L^ were more resistant to A22 than RMR cells to 1 µg/mL A22, suggesting that these MreC and MreD mutants increased the integrity of the Rod complex. RodZ and RMR cells producing PBP2^A201V^ or PBP2^R234L^ showed similar A22‐sensitivity to RodZ or RMR cells; and RodZ or RMR cells producing PBP2^T52I^, PBP2^I59S^, PBP2^V227L^, RodA^A234T^, or RodA^K243N^ were more resistant to 1 or 5 µg/mL A22 than RodZ or RMR cells (Figure 5b), supporting the idea that these mutations increased the integrity of the Rod complex or the activity of the Rod complex. If these mutations increase the integrity of the Rod complex and therefore peptidoglycan synthesis activity is increased compared with that of RMR cells, the suppressor cells may also change the sensitivity to mecillinam, which specifically binds to PBP2. Thus, we examined the sensitivity of the suppressor cells to mecillinam (Figure 5). RMR cells producing MreB^E122D^, MreB^R124S^, MreB^A125V^, MreB^E137G^, MreD^F123L^, all suppressor mutants in PBP2 except PBP2^A201V^, RodA^A234T^, or RodA^K243N^ were slightly more resistant to 0.1 µg/mL mecillinam than RMR cells. These suppressor cells may have increased peptidoglycan synthesis activity compared with that of RMR. It was shown that RodA^A234T^ has an increased activity of the Rod complex (Rohs et al., 2018). However, RodZ cells producing MreB^R124L^ were more sensitive to mecillinam than WT cells. RMR cells producing MreC^S153I^ were more sensitive to mecillinam than RMR cells producing MreD^F123L^, suggesting that MreD^F123L^ functions as a stronger suppressor of RMR than MreC^S153I^. The morphology of RMR cells producing MreD^F123L^ is more similar to rod‐shaped WT than that of RMR cells producing MreC^S153I^.

### 3.5 Peptidoglycan structure revealed by QFDE‐EM

**Figure 6:**
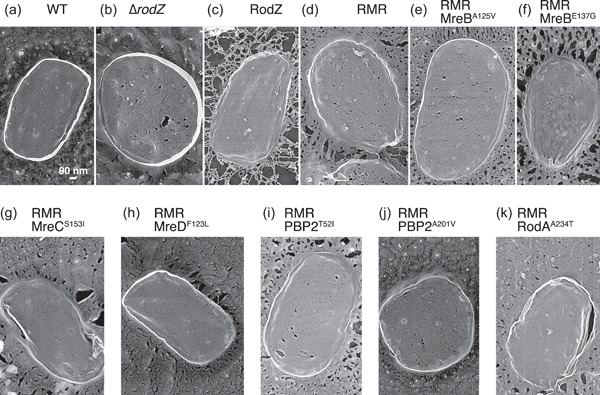
Structures of peptidoglycan. (a–k) Peptidoglycan purified from the indicated strain was observed by quick freeze, deep‐etch, and electron microscopy. Representative pictures are shown.

**Figure A7:**
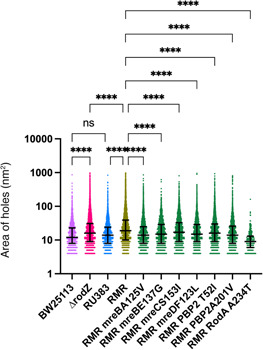
Size of holes in peptidoglycan. Violin plots showing the distribution of the size of holes in peptidoglycan purified from the indicated strains. Distribution of the size and number of holes in peptidoglycan purified from each strain. The size and number of holes in the images of the three peptidoglycans were quantified. Average and standard deviation (SD) are shown. *p* Values were determined by unpaired *T* test. ns: *p* > 0.05, *p ≤ 0.05, **p ≤ 0.01, ***p ≤ 0.001, ****p ≤ 0.0001.

So far, we have shown that RMR cells were abnormal in shape and that RMR formed aberrant clusters; the suppressors restored these phenotypes. We hypothesized that the aberrant Rod complexes containing RMR were unable to synthesize peptidoglycan properly. If the correct peptidoglycan synthesis does not take place as a result of the reduced peptidoglycan synthesis activity of Rod complexes containing RMR, then the resulting peptidoglycan structures would be abnormal. We purified peptidoglycan from various strains and observed their structures using QFDE‐EM (Tulum et al., 2019) to directly observe peptidoglycan purified from WT and RMR. Peptidoglycan purified from BW25113 (WT) and RU383 (sfGFP‐RodZ) cells showed a relatively homogeneous structure (Figure 6a,c, Figure A7, and Table 6). Many small holes were observed, as previously observed (Demchick & Koch, 1996; Pasquina‐Lemonche et al., 2020). There was no significant difference in the pore size of the peptidoglycan of BW25113 (WT) (19.8 ± 28.6 nm^2^) and RU383 (RodZ) (21.2 ± 31.7 nm^2^) (*p* = 0.0066). The peptidoglycan purified from the RMR cells clearly had larger holes (42.2 ± 81.0 nm^2^), and the number of holes was higher than that of the peptidoglycan purified from WT cells (Figure 6d, Figure A7 and Table 6), suggesting that the Rod complexes containing RMR synthesize aberrant peptidoglycan, leading to the abnormal shape. The pore size of the peptidoglycan of ∆*rodZ* (30.0 ± 51.0 nm^2^) was smaller than that of RMR (Figure 6b,d and Figure A7) but larger than that of the wild strain. We observed the structure of peptidoglycan purified from RMR cells carrying a suppressor mutation. The number of holes was clearly reduced compared with that of RMR peptidoglycan but was still higher than that in WT peptidoglycan (Figure 6e–k, Figure A7, and Table 6). The size of these holes except for RMR RodA^A234T^ was almost the same as that in the WT peptidoglycan (Figure A7 and Table 6). These results suggest that, in the suppressor strains, the activity of the Rod complex containing RMR was increased by strengthening the protein–protein interactions within the Rod complex, or by unknown mechanisms, thus allowing the synthesis of the correct peptidoglycan.

**Table 6:** The number and size of holes in purified peptidoglycan.

Peptidoglycan purified from	Number^a^	Size (nm^2^)^b^
WT (RodZ)	3353	19.7 ± 28.6
∆*rodZ*	9809	30.0 ± 51.0
WT (sfGFP‐RodZ)	3660	21.7 ± 31.7
RMR	7574	42.1 ± 81.1
RMR MreB^A125V^	4462	23.6 ± 36.7
RMR MreB^E137G^	1917	23.9 ± 32.4
RMR MreC^S153I^	3385	29.3 ± 42.0
RMR MreD^F123L^	3722	26.4 ± 40.9
RMR PBP2^T52I^	3718	28.4 ± 47.2
RMR PBP2^A201V^	2108	25.8 ± 40.9
RMR RodA^A234T^	347	11.7 ± 11.5

^a^ Sum of the holes of three purified peptidoglycan surfaces.

^b^ Mean ± standard deviation (nm^2^) of the size of the holes of three purified peptidoglycan surfaces.

### 3.6 Chemical structures of peptidoglycan

**Figure 7:**
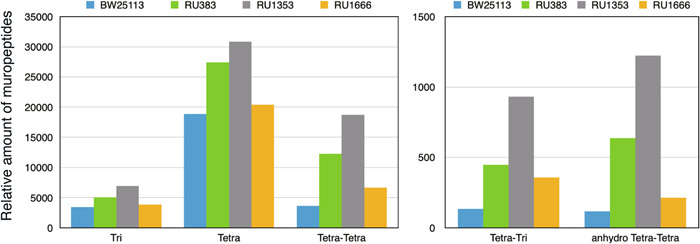
Compositions of muropeptides. Muropeptides were purified from BW25113 (RodZ), RU383 (sfGFP‐RodZ), RU1353 (sfGFP‐RMR), and RU1666 (sfGFP‐RMR MreB^A125V^), respectively. The experiment was conducted twice, with essentially the same results.

We showed by using QFDE‐EM that the overall abnormal structures of the peptidoglycan from cells producing RMR reverted to the normal peptidoglycan structure with each suppressor mutation (Figure 6). We next examined how the structures of muropeptides of RMR and suppressors differ chemically from that of WT by using LC/MS analysis of purified peptidoglycan. Here, we used BW25113 (WT) as a control strain and analyzed RU1666 (sfGFP‐RMR MreB^A125V^) as a suppressor strain. Tri, Tetra, Tetra‐Tetra, Tetra‐Tri, and anhydro Tetra‐Tetra muropeptides were the most abundant in RU1353 compared with other strains (Figure 7). Tetra‐Tetra is a structure normally found in peptidoglycan. Therefore, it appears that the Rod complex is more active in RU1353 compared with RU383, contrary to our previous conclusion. On the other hand, other muropeptides are mainly present during and after cell wall repair, suggesting that peptidoglycan of RU1353 was damaged and repaired much compared with other strains. It was shown that β‐lactam antibiotics which inhibited the activity of PBPs induced a futile cycle of cell wall synthesis and degradation (Cho et al., 2014). Thus, the increase in Tetra‐Tetra muropeptides in RU1353 was not simply an increase in the activity of the Rod complex but rather suggests that a futile cycle of cell wall synthesis and degradation was induced in RU1353. Furthermore, a comparison of the muropeptide composition of BW25113 and RU383 showed that each muropeptide was more abundant in RU383 (Figure 7). The difference between these strains is whether or not sfGFP is fused to the N‐terminus of RodZ. The results, therefore, suggest that, although there are no major differences in the growth rate, morphology, or overall structure of the peptidoglycan between the two strains (Figures A1 and A7), there are differences in the chemical structure of the peptidoglycan. In other words, fusing sfGFP to RodZ may reduce the activity of the Rod complex. Interestingly, the muropeptide compositions of RU1616 (sfGFP‐RMR MreB^A125V^) were rather closer to BW25113 (WT) than to RU383 (sfGFP‐RodZ). Therefore, the MreB^A125V^ mutation suppresses not only the reduced function of the Rod complex by RMR but also the reduced function of the Rod complex by the fusion of sfGFP.

## 4 DISCUSSION

Many of the factors in the Rod complex, such as MreB and PBP2, are essential for viability in rich medium (Bendezú & de Boer, 2008), but RodZ is not (Bendezú et al., 2009; Shiomi et al., 2008). *E. coli* ∆*rodZ* cells are spherical or oval (Bendezú et al., 2009; Shiomi et al., 2008). An analysis of the function of each region of the transmembrane protein RodZ was performed previously (Bendezú et al., 2009; Shiomi et al., 2008). The N‐terminal cytoplasmic region interacts with MreB, and the C‐terminal periplasmic region interacts with RodZ, MreC, MreD, and PBP2 (Bendezú et al., 2009; van den Ent et al., 2010; Ikebe et al., 2018). In addition, the interaction between MreC and PBP2 may be important for the activation of the Rod complex (Contreras‐Martel et al., 2017; Liu et al., 2020; Rohs et al., 2018; Rohs et al., 2021). Therefore, since the RodZ protein interacts with the cytoskeletal protein MreB in the cytoplasmic domain to stabilize the Rod complex, and also interacts with the peptidoglycan synthesis enzyme PBP2 in the periplasmic domain, possibly promoting peptidoglycan synthesis, the transmembrane region of RodZ may play an important role in connecting these two interactions. Consistently, most of the suppressor mutations that restored the slow‐growth phenotype and morphological abnormality of ∆*rodZ* were found in the components of the Rod complex (Shiomi et al., 2013). We constructed an RMR‐producing strain to elucidate the function of the transmembrane region of RodZ and found that RMR cells are not as morphologically abnormal as ∆*rodZ* cells, but they are also not the same as WT cells. Furthermore, the growth of RMR cells was slower than that of WT. Presumably, these various abnormalities were caused by the lower activity of the Rod complexes containing RMR to synthesize peptidoglycan compared with that of Rod complexes containing WT RodZ. The complex formation of Rod complexes containing RMR was not normal, which resulted in the lower activity of Rod complexes containing RMR. The transmembrane region of RodZ is essential for the correct role of RodZ, although it can be replaced by MalF^17‐39^ for anchoring RodZ to the membrane. Perhaps the replacement of the transmembrane region of RodZ with MalF^17‐39^ does not preserve the correct structure of RodZ. To further investigate the role of the transmembrane domain of RodZ, we isolated suppressors of RMR cells, hoping to find mutations within the RMR molecule, but no such mutations were found. The absence of suppressor mutations in the cytoplasmic interaction site with MreB and the periplasmic interaction site with PBP2 in the RMR molecule suggests that a single mutation in one of these regions is not sufficient for suppression and that the transmembrane region of RodZ is important for the correct connection between the cytoplasmic and periplasmic regions in the Rod complex. Instead, most of the mutations were found in the Rod complex, such as *mreB* and *mrdA* (encoding PBP2). Some of these were already isolated as suppressors of ∆*rodZ* cells (Shiomi et al., 2013). When these mutation sites were mapped onto the structure of each protein, they were located at protein–protein interaction sites. MreB^R124^ and MreB^A125V^ are located at the interface between the MreB filaments (van den Ent et al., 2014). It was shown that MreC is important for inducing conformational changes in PBP2, and growth defects caused by MreC^G156D^ were suppressed by PBP2^T52A^, PBP2^L61R^, and RodA^A234T^ (Rohs et al., 2018). Furthermore, it was shown that PBP2^L61R^ and RodA^A234T^ increased peptidoglycan synthesis activity (Rohs et al., 2018). We isolated *mreC* ^*S153I*^, *mrdA* ^*T52I*^, *mrdA* ^*I59S*^, *mrdB* ^*A234T*^, and *mrdB* ^*K243N*^ as suppressor mutations in RMR cells. These are located in the same regions as MreC^G156^, PBP2^T52A^, PBP2^L61R^, and RodA^A234T^. Therefore, it is reasonable to assume that the increased peptidoglycan synthesis activity of these mutants suppressed the lower activity of RMR. It was shown that MreD also interacts with PBP2 and MreD negatively affects the interaction between MreC and PBP2. Thus, the MreD^F123L^ mutant may change the interaction between MreC and PBP2, hence the activity of the Rod complex. It should be noted that there are some types of suppression of cell length. For example, the length of sfGFP‐RodZ cells producing MreB^R124L^, MreB^R124S^, or PBP2^V227L^ was close to that of BW25113 (WT) while the length of sfGFP‐RMR producing MreB^R124L^, MreB^R124S^, or PBP2^V227L^ was close to that of RU383 (sfGFP‐RodZ) although they are significantly statistically different from that of RU383. On the other hand, the length of sfGFP‐RMR cells producing MreB^E122D^, PBP2^T52I^, RodA^A234T^, or RodA^K243N^ was close to that of RU383 while these mutations did not largely affect the length of RU383. In conclusion, the role of RodZ, especially its transmembrane domain, is to optimize the interactions of the components in the Rod complex, thus regulating the activity of the complex.

Peptidoglycan has been observed by EM and AFM, and its structures have been reported (de Pedro et al., 1997; Elsbroek et al., 2023; Gan et al., 2008; Pasquina‐Lemonche et al., 2020; Salamaga et al., 2021; Tulum et al., 2019; Turner et al., 2018). In particular, QFDE‐EM is an excellent tool for visualizing the structure of peptidoglycan with high resolution. Using this method, the cell surface of *B. subtilis* and the outer membrane vesicles of *E. coli* and their formation processes have been observed in detail (Ojima et al., 2021; Tahara & Miyata, 2023; Tulum et al., 2019). The relationship between the activity of the Rod complex and the overall structure of the peptidoglycan has been controversial. Turner et al. showed that the pore sizes of peptidoglycan are not different even after A22, an inhibitor of MreB (Iwai et al., 2002), was added to cells (Turner et al., 2018) while Elsbroek et al., showed that peptidoglycan became less dense when cells were treated with β‐lactam antibiotics (Elsbroek et al., 2023). We isolated peptidoglycan from WT, RMR, and suppressor cells and visualized their structures at high resolution by QFDE‐EM. Very small holes were observed in the peptidoglycan purified from WT. In our observation, the average size of the hole was ~20 nm^2^. It has been reported that the radius of the hole of *E. coli* peptidoglycan is 2.06 nm (approximately 13 nm^2^ in area) (Demchick & Koch, 1996). In other reports estimated by AFM, the diameter of the hole of *E. coli* peptidoglycan is 10 nm (approximately 79 nm^2^ in area) (Elsbroek et al., 2023; Turner et al., 2013, 2018). In the computer simulations, the maximum pore radius averaged over time was 2.05–2.44 nm (approximately 13–19 nm^2^ in area) (Gumbart et al., 2014). The diameter of more than half of the holes in the peptidoglycan of *Staphylococcus aureus* was reported to be 6.2 nm (approximately 30 nm^2^ in area) based on the results of AFM observation (Pasquina‐Lemonche et al., 2020). Our observations are in good agreement with these previous results. On the other hand, the number and size of holes in the peptidoglycan purified from RMR are larger compared with those from the WT cells. It is assumed that hydrolysis of peptidoglycan to incorporate the new peptidoglycan occurs normally in RMR cells so that peptidoglycan in cells with low synthetic activity (RMR cells) would have large holes. Interestingly, both the number and the size of the holes in the peptidoglycan purified from the suppressor strains were reduced compared with those of the peptidoglycan purified from RMR. Perhaps the activity of the Rod complex was increased in the suppressors, allowing it to synthesize peptidoglycan correctly. Turner et al. (2018) reported that no change in the pore size of peptidoglycans was observed with the addition of A22. On the other hand, Elsbroek et al. (2023) reported that the addition of β‐lactam antibiotics made peptidoglycans less dense, that is, the pores became larger, in which the authors concluded that their results are consistent with the mechanism of action of β‐lactam antibiotics to inhibit peptide cross‐linking. Although we do not know why these observations using AFM obtained different results, our results are consistent with those of Elsbroek et al. (2023) and are therefore consistent with our conclusion that the RMR‐producing strain probably has reduced peptidoglycan synthesis activity. Furthermore, Salamaga et al. (2021) reported that peptidoglycan purified from *S. aureus* cells treated with methicillin or vancomycin had larger holes than those purified from nontreated cells, using AFM. Since methicillin and vancomycin inhibit peptidoglycan synthesis, it was concluded that the holes were generated because the hydrolytic activity of the peptidoglycan exceeded that of its synthesis. This result was in good agreement with our observations. We conclude that the Rod complex may be a determinant not only for the whole shape of peptidoglycan and cell morphology but also for its highly dense structure to support the mechanical strength of the cell wall.

Our LC/MS analysis of purified peptidoglycan revealed that Tri, Tetra, Tetra‐Tetra, Tetra‐Tri, and anhydro Tetra‐Tetra muropeptides were the most abundant in RU1353 compared with other strains. The increase in Tetra‐Tetra muropeptides, which are normally present in peptidoglycan, in RU1353 was not simply an increase in the activity of the Rod complex but rather suggests that a futile cycle of cell wall synthesis and degradation was induced in RU1353. The decrease in the activity of the Rod complex would lead to peptidoglycan damage and subsequent repair of peptidoglycan. This is consistent with the results of electron microscopic observation of peptidoglycan. Furthermore, the suppressor mutation indeed suppressed the reduced activity of the Rod complex containing RMR. However, this analysis also yielded an unexpected result: although fusing sfGFP to RodZ did not significantly affect the growth rate, cell morphology or overall structure of the peptidoglycan in cells producing sfGFP‐RodZ (Figures A1 and A7), the compositions of the muropeptide were different (Figure 7). The results suggest that the activity of the Rod complex containing sfGFP‐RodZ is slightly reduced compared with that of the wild‐type strain. Observations of *E. coli* cells producing MreB fused with fluorescent proteins have been also reported (Bendezú et al., 2009; Ouzounov et al., 2016). For example, MreB‐msfGFP^SW^ complemented the *mreB* defect and showed normal morphology, whereas MreB‐mGFPmut3^SW^ failed to complement the *mreB* defect and showed abnormal morphology (Ouzounov et al., 2016). Ouzounov et al. showed that the growth rate of cells producing MreB‐msfGFP^SW^ was comparable with that of WT cells while the cell width of cells producing MreB‐msfGFP^SW^ was ~5% wider than that of WT cells. To our knowledge, no muropeptides have been analyzed in cells expressing a fusion protein between a component of the Rod complex and a fluorescent protein. Therefore, this study is the first to show that fusing fluorescent proteins affects the activity of the Rod complex. However, even in such cells, the growth rate and morphology were not significantly affected. Thus, *E. coli* has the robustness to retain its overall structure even if the peptidoglycan structure is somewhat changed.

In this work, we visualized the peptidoglycan of *E. coli* at high resolution without chemical treatment and analyzed the muropeptide compositions. In the future, by combining these methods, we would like to analyze mutant strains of factors involved in peptidoglycan synthesis, degradation, and repair to gain a macroscopic understanding of how each protein plays a role in maintaining the peptidoglycan structure.

## AUTHOR CONTRIBUTIONS

**Risa Ago**: Conceptualization (equal); data curation (equal); formal analysis (equal); writing—original draft (equal). **Yuhei O. Tahara**: Data curation (equal); formal analysis (equal); investigation (equal); writing—original draft (equal). **Honoka Yamaguchi**: Formal analysis (equal); investigation (equal). **Motoya Saito**: Formal analysis (equal); investigation (equal). **Wakana Ito**: Formal analysis (equal); investigation (equal). **Kaito Yamasaki**: Formal analysis (equal); investigation (equal). **Taishi Kasai**: Formal analysis (equal); methodology (equal). **Sho Okamoto**: Data curation (equal); formal analysis (equal); investigation (equal); writing—original draft (equal). **Taiki Chikada**: Investigation (equal). **Taku Oshima**: Data curation (equal); formal analysis (equal); funding acquisition (equal); investigation (equal); writing—review and editing (equal). **Issey Osaka**: Data curation (equal); formal analysis (equal); funding acquisition (equal); investigation (equal); writing—review and editing (equal). **Makoto Miyata**: Data curation (equal); formal analysis (equal); funding acquisition (equal); writing—original draft (equal). **Hironori Niki**: Conceptualization (equal); data curation (equal); formal analysis (equal); writing—original draft (equal). **Daisuke Shiomi**: Conceptualization (lead); data curation (lead); funding acquisition (lead); investigation (lead); methodology (lead); project administration (lead); supervision (lead); writing—original draft (lead); writing—review and editing (lead).

## CONFLICT OF INTEREST STATEMENT

None declared.

## ETHICS STATEMENT

None required.

## Data Availability

All data are provided in full in the results section and the appendix of this paper.
